# Cancer-Related Internet Use and Its Association With Patient Decision Making and Trust in Physicians Among Patients in an Early Drug Development Clinic: A Questionnaire-Based Cross-Sectional Observational Study

**DOI:** 10.2196/10348

**Published:** 2019-03-14

**Authors:** Goldy C George, Eucharia C Iwuanyanwu, Adrianna S Buford, Sarina A Piha-Paul, Vivek Subbiah, Siqing Fu, Daniel D Karp, Shubham Pant, Christina O Hinojosa, Kenneth R Hess, Charles S Cleeland, Elmer V Bernstam, Funda Meric-Bernstam, David S Hong

**Affiliations:** 1 Department of Investigational Cancer Therapeutics The University of Texas MD Anderson Cancer Center Houston, TX United States; 2 Department of Biostatistics The University of Texas MD Anderson Cancer Center Houston, TX United States; 3 Department of Symptom Research The University of Texas MD Anderson Cancer Center Houston, TX United States; 4 School of Biomedical Informatics The University of Texas Health Science Center at Houston Houston, TX United States

**Keywords:** cancer, clinical trial, doctor, early-phase clinical trial, internet, patient, physician, patient-physician relationship, symptoms

## Abstract

**Background:**

The role of cancer-related internet use on the patient-physician relationship has not been adequately explored among patients who are cancer-related internet users (CIUs) in early-phase clinical trial clinics.

**Objective:**

We examined the association between cancer-related internet use and the patient-physician relationship and decision making among CIUs in an early drug development clinic.

**Methods:**

Of 291 Phase I clinic patients who completed a questionnaire on internet use, 179 were CIUs. Generations were defined by the year of patient’s birth: “millennials” (after 1990) and “Generation X/Y” (1965-1990) grouped as “Millennials or Generation X/Y”; “Baby Boomers” (1946-1964); and “Greatest or Silent Generation” (1945 and earlier). Statistical analyses included the Wilcoxon matched-pairs signed-rank test and the Mann-Whitney U test.

**Results:**

CIUs were 52% (94/179) female, 44% (78/179) were older than 60 years, and 60% (108/179) had household incomes exceeding US $60,000. The sources of information on cancer and clinical trials included physicians (171/179, 96%), the internet (159/179, 89%), and other clinical trial personnel (121/179, 68%). For the overall sample and each generation, the median values for trust in referring and Phase I clinical trial physicians among early drug development clinic CIUs were 5 on a 0-5 scale, with 5 indicating “complete trust.” CIUs’ trust in their referring (5) and phase 1 (5) physicians was higher than CIUs’ trust in Web-based cancer-related information (3; *P*<.001 for both). CIUs who reported visiting the National Cancer Institute (NCI) website, NCI.org, to learn about cancer reported higher levels of trust in Web-based cancer-related information than CIUs who did not use the NCI website (*P*=.02). Approximately half of CIUs discussed internet information with their doctor. Only 14% (23/165) of CIUs had asked their physician to recommend cancer-related websites, and 24% (35/144) of CIUs reported at least occasional conflict between their physician’s advice and Web-based information.

**Conclusions:**

Despite the plethora of websites related to cancer and cancer clinical trials, patients in early-phase clinical trial settings trust their physicians more than Web-based information. Cancer-related organizations should provide regularly updated links to trustworthy websites with cancer and clinical trial information for patients and providers and educate providers on reliable cancer websites so that they can better direct their patients to appropriate internet content.

## Introduction

Early-phase clinical trials form a critical link between preclinical testing of novel therapies and Food and Drug Administration approval of these agents [1]. Patients in early-phase oncology clinical trials tend to have advanced cancer that is refractory to standard therapies. Given the complexities of early-phase clinical trials [1] and the advanced nature of the disease being treated, patients in early drug development clinics need accurate information when they consider enrollment or continued treatment in clinical trials. Information for early-phase clinical trial patients is especially important in the light of the suggestion that some patients may join early-phase clinical trials without fully understanding all of the goals of Phase I trials (particularly goals related to the research aspects of an early-phase clinical trial, such as dose escalation and estimation of maximum tolerated dose) [2]. Also, patients may sometimes join clinical trials with unrealistic expectations of personal disease-related benefits from the trial, under a condition termed as the therapeutic misconception [2].

The internet is often used as a source of cancer-related information by patients with cancer [3], including early-phase clinical trial clinic patients [4]. The information found on the internet may be a source of empowerment for some patients, but it may also present conflicting information, leaving some patients anxious or confused [4,5]. Also, with the mainstreaming of social networking sites, patients are able to form a circle of faceless friends in chat rooms, through blogs, and in Web-based communities dedicated to people like themselves. For example, the use of internet cancer support groups has increased [6]. Thus, it has been stated that the internet has the potential to redefine patient-physician relationship due to the vast amounts of information on the internet that are accessible to patients [7,8]. Also, patients may bring internet-derived cancer- and clinical trial-related information to consultations with their oncologist [3] to supplement, confirm, or refute information provided by their physician. Alternatively, patients may seek out vast amounts of cancer- and treatment-related information to promote a trusting and therapeutic doctor-patient relationship [9] (relating to the concept of psychological autonomy). While several models (including paternalistic, informative, interpretive, and deliberative models) [10-12] have been proposed to understand or explain the doctor-patient relationship and the level of patient involvement in the treatment decision-making process, the patient-physician relationship and related aspects have not been adequately explored among patients in early drug development clinic settings who use the internet for cancer-related purposes. The purpose of this study was to examine cancer-related internet users (CIUs) in an early drug development clinic, factors related to the patient-physician relationship (eg, patients’ trust in referring and clinical trial physicians), and decision making (eg, patients’ decisions, such as selecting the treating hospital and joining a clinical trial). This study ties in with the goals of the National Cancer Moonshot, which seeks to increase patient access to quality cancer care and increase clinical trial participation.

## Methods

### Procedure

Patients were recruited from the Clinical Center for Targeted Therapy (CCTT), the Phase I clinical trials clinic at The University of Texas MD Anderson Cancer Center. CCTT is one of the largest early-phase clinical trial centers in the world. CCTT patients have advanced solid tumors or lymphomas and are self-referred or referred by their primary oncologist. Phase I or CCTT patients were approached with a standardized script and requested to complete an anonymous questionnaire as they waited for their scheduled clinical appointment. Patients were asked to deposit their completed questionnaire in a locked box, rather than to return it to a person, in order to ensure the anonymity of the patient completing the survey.

### Patients

Patients who had a diagnosis of advanced cancer, were able to read and understand English, and were aged ≥18 years were eligible for the study. Patients who had severe visual or cognitive impairment that would interfere with the ability to complete the questionnaire were excluded. The study was approved by the Institutional Review Board of MD Anderson, and all participants provided informed consent.

### Materials

The questionnaire was developed by an investigative team with expertise in early-phase clinical trials and research questionnaire development methodology [4,13]. Questions were based on extant literature relating to internet use in patients with cancer [14-17] or developed de novo for our study, taking into account the unique nature of the phase I clinic’s patient population [4]. The questionnaire was pilot-tested among 18 patients from the early-phase clinical trials clinic, and feedback on the ease of understanding, readability, and applicability of the context to patients with advanced cancer was elicited [4]. Based on patients’ feedback during the pilot test, the survey instrument was further revised. The final survey instrument was thus tailored to the early-phase clinical trials context. The questionnaire included questions on demographics, cancer- related internet use, trust in physicians, and patient decision making related to whether they use the internet to make decisions about where to be treated and whether to join a trial. For example, trust in a referring physician was assessed using the question, “How much do you trust your referring physician?” Trust in Web-based cancer information was assessed with “How much do you trust Web-based cancer-related information?” Trust in referring and Phase I physicians and trust in Web-based cancer-related information were measured using six-point Likert-type scales where 0=none and 5=complete trust.

### Statistical Analysis

SPSS v23 (IBM Corp) was used for analyses. Generational differences have been shown to be associated with certain aspects of cancer internet use patterns [18]. Thus, patients were grouped by generation based on year of birth: “Millennials” (after 1990) and “Generation X/Y” (1965-1990) combined as “Millennials or Generation X/Y”; “Baby Boomers” (1946-1964); and “Greatest or Silent Generation” (1945 and earlier). Box plots were used to graphically represent the distributions (including medians and interquartile ranges) of the variables of trust in referring and Phase I physicians and trust in Web-based cancer-related information for the overall sample and by generation. The Wilcoxon matched-pairs signed-rank test was used to compare CIUs’ trust in physicians with their trust in Web-based cancer-related information. The Mann-Whitney U test was used to compare CIUs’ trust in their referring or Phase I physician and trust in Web-based cancer-related information based on whether they used selected websites (eg, National Cancer Institute [NCI] website) to learn about cancer or clinical trials. Significance was set at an alpha level of.05, and all tests were two-tailed.

## Results

### Patient Characteristics

The questionnaire was completed by 291 patients in the phase I clinic (CCTT), of whom 179 (179/291, 62%) were CIUs; these CIUs are the focus of this manuscript. CIUs were defined as those who had indicated a response of “Yes” to the question “Do you access the internet for cancer-related purposes (for example, for cancer- or clinical trial-related information or for emotional or social support for your cancer)?” Characteristics of CIUs are included in Table 1.

### Sources of Information on Cancer and Clinical Trials for Patients

Among CIUs in the Phase I clinical trials clinic, 96% (171/179) relied on physicians as a source of information on cancer and clinical trials and 89% (159/179) used the internet for cancer-related and clinical trial-related information. Other sources of information that CIUs used to learn about cancer and cancer treatment or clinical trials included other clinical trial personnel, such as nurses or physician assistants (121/179, 68%); information pamphlets from the treating hospital, the NCI, the American Cancer Society, the American Society of Clinical Oncology, or other cancer-related organizations (83/179, 46%); other patients (64/179, 36%); books (47/179, 26%) and magazines (35/179, 20%); scientific journals (33/179, 18%); and smartphone apps (8/179, 5%). Multimedia Appendix 1 shows the sources of information on cancer and cancer treatment (including clinical trials) for the overall sample of patients in the early drug development (Phase I clinic; N=291).

### Internet and Patient Decision Making Related to the Selection of Treating Hospital and Clinical Trials Clinic

The major drivers of where CIUs decided to receive their cancer care were the reputation of the hospital and referring physician recommendations (Table 2). Nearly 58% (99/171) of CIUs reported that the internet had not influenced their decision to come to the treating hospital in any way; the remaining 42% (72/171) of CIUs indicated that the internet had influenced their decision to come to the treating hospital. Most CIUs indicated that the internet had not influenced their decision to visit the Phase I clinic at the treating hospital (134/165, 81%) or their decision to enroll in a Phase I clinical trial (129/160, 81%). Nearly 90% (149/168, 89%) of CIUs stated that the major determinant of their choice of clinical trial physician was based on their referring physician’s recommendation.

**Table 1:** Characteristics of cancer-related internet users in an early drug development clinic (N=179).

Characteristics		Internet users, n (%)
**Generation**		
	Millennials or Generation X/Y	49 (24)
	Baby Boomers	89 (47)
	Greatest or Silent	41 (29)
**Gender**		
	Male	85 (48)
	Female	94 (53)
**Race or ethnicity**		
	Nonminority	152 (85)
	Minority	27 (15)
**Tumor type**		
	Lung	22 (12)
	Colorectal	20 (11)
	Melanoma	19 (11)
	Head and neck	16 (9)
	Ovarian	16 (9)
	Breast	15 (8)
	Sarcoma	10 (6)
	Prostate	9 (5)
	Endometrial	9 (5)
	Pancreatic	6 (3)
	Others^a^	37 (21)

^a^Included thyroid (n=6), lymphoma (n=3), hepatocellular (n=3), cervical (n=3), kidney (n=2), brain (n=2), and other tumor types (n=18).

**Table 2:** Decision making related to cancer treating hospital and early-phase clinical trial enrollment among cancer-related internet users in an early drug development clinic.

Questionnaire item		Overall (N=179), n (%)	Millennials or Generation X/Y (n=49), n (%)	Baby Boomers (n=89), n (%)	Greatest or Silent (n=41), n (%)
**How did you decide where to get your cancer care?**					
	Reputation of cancer care organization/hospital	112/174 (64)	35/48 (73)	47/85 (55)	30/41 (73)
	Physician recommendation	111/174 (64)	30/48 (63)	57/85 (67)	24/41 (59)
	Family or friend recommendation	42/174 (24)	12/48 (25)	19/85 (22)	11/41 (27)
**Did the information on the Internet influence your decision to come to the treating hospital?**					
	No, not at all	99/171 (58)	21/48 (44)	53/83 (64)	25/40 (63)
	Yes, absolutely	45/171 (26)	14/48 (29)	22/83 (27)	9/40 (23)
	Yes, somewhat	27/171 (16)	13/48 (27)	8/83 (10)	6/40 (15)
**Did the information on the Internet influence your decision to come to the Phase I clinic at the treating hospital?**					
	No, not at all	134/165 (81)	38/47 (81)	63/81 (78)	33/37 (89)
	Yes, somewhat	15/165 (9)	4/47 (9)	8/81 (10)	1/37 (3)
	Yes, absolutely	16/165 (10)	5/47 (11)	10/81 (12)	3/37 (8)
**Did the information on the Internet influence your decision to enroll on a Phase I clinical trial?**					
	No, not at all	129/160 (81)	35/45 (78)	62/79 (79)	32/36 (89)
	Yes, somewhat	14/160 (9)	4/45 (9)	9/79 (11)	2/36 (6)
	Yes, absolutely	17/160 (11)	6/45 (13)	8/79 (10)	2/36 (6)
**How did you decide which clinical trial physician to use?**					
	Referring physician recommendation	149/168 (89)	44/47 (94)	71/83 (86)	34/38 (90)
	Assigned by the treating hospital	15/168 (9)	1/47 (2)	10/83 (12)	4/38 (11)
	Family or friend recommendation	6/168 (4)	3/47 (6)	1/83 (1)	2/38 (5)
	Internet	4/164 (2)	1/47 (2)	4/83 (5)	1/38 (3)

### Patient Discussions of Internet Information With Their Physician

Approximately half of the CIUs indicated that they had discussed or planned to discuss internet information with their doctor (Table 3). Among CIUs who answered “Yes” to the question “Have you discussed/do you plan to discuss Internet information with your doctor?”, reasons cited for discussing internet information with their physician included wanting to educate themselves (69/86, 80%), to be proactive to improve their health (65/86, 76%), to obtain their physician’s expertise (62/86, 72%), to educate their caregiver or family member (37/86, 43%), to meet an emotional need (10/86, 12%), or other reasons (to “clarify confusing information”, “discuss options”, “make sure important information is correct”, 3/86, 4%). For CIUs who answered “No” to the question “Have you discussed/do you plan to discuss internet information with your doctor?”, reasons for not doing so included trusting their physician to make the best choice for them (31/68, 46%), not feeling a need to clarify anything with their physician (22/68, 32%), believing that the information was not relevant to their situation or disease (5/68, 7%), forgetting to mention the information (5/68, 7%), not having enough time with physician (4/68, 6%), not wanting to bother the doctor (4/68, 6%), or other reasons (including “they already know”, 2/68, 3%).

### Comparison of Patients’ Trust in Referring and Phase I Physicians and Their Trust in Web-based Cancer Information

**Figure 1:**
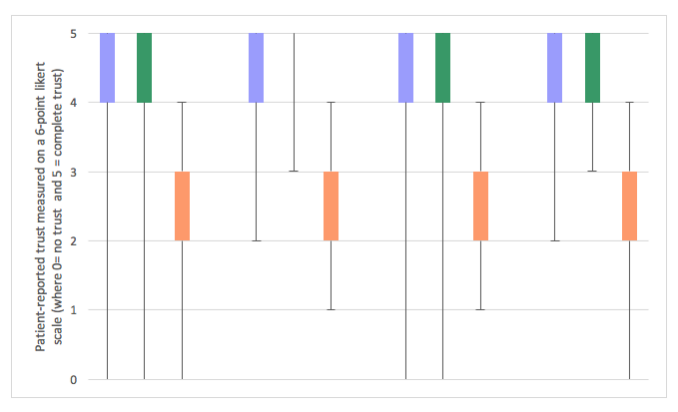
Box plots showing the distribution of trust in referring physicians, early-phase clinical trial physicians, and Web-based cancer-related information for cancer-related internet users (CIUs) in an early drug development clinic. Purple boxes indicate trust in referring physician, green boxes indicate trust in Phase I clinical trial physician, and orange boxes indicate trust in Web-based cancer-related information. In the overall sample of CIUs and within each generation, CIUs reported significantly higher trust in their referring or Phase I physician than trust in Web-based cancer-related information.

In the overall sample and in each generation, median values for trust in the referring and Phase I clinical trial physicians among CIUs were 5 on a 0-5 scale, with 5 indicating “complete trust” (Figure 1). Trust in referring or Phase I physician did not differ significantly between CIUs who reported accessing the internet at least sometimes through smartphone or cell phone for cancer or clinical trial information and CIUs who reported that they never or rarely did so (*P*>.05). Also, CIUs’ trust in their referring or phase I physician did not vary by CIUs self-reported rating of their symptoms of feeling depressed or anxious, pain, tiredness or fatigue, or of their experience of difficulty moving around (*P*>.05).

**Table 3:** Impact of the internet on the patient-physician relationship among cancer-related internet users in an early drug development clinic.

Characteristics		Overall (N=179), n (%)	Millennials or Generation X/Y (n=49), n (%)	Baby Boomers (n=89), n (%)	Greatest or Silent (n=41), n (%)
**Have you discussed/do you plan to discuss Internet information with your doctor?**					
	Yes	87/167 (52)	26/46 (56)	49/82 (60)	12/39 (31)
	No	80/167 (48)	20/46 (44)	33/82 (40)	27/39 (69)
**How do you verify that information from the Internet is correct?**					
	I ask my doctor	111/156 (71)	33/46 (72)	52/73 (71)	26/37 (70)
	I ask the PA, APN, or other members of the clinical team	64/156 (41)	14/46 (30)	36/73 (49)	14/37 (38)
	If I see it in multiple places I believe it is true	34/156 (22)	14/46 (30)	13/73 (18)	7/37 (19)
	I don’t verify that things I read online are true	14/156 (9)	3/46 (7)	6/73 (8)	5/37 (14)
	Other (source credibility, I check other sources for data and evidence, I visit only reliable sites, sometimes I just take a chance)	8/156 (5)	2/46 (4)	6/73 (8)	0/37 (0)
**Do you trust your physician’s advice over the information found on the Internet?**					
	Yes	158/163 (97)	43/44 (98)	79/82 (96)	36/37 (97)
	No	5/163 (3)	1/44 (2)	3/82 (4)	1/37 (3)
**If you trust your physician’s advice more, what are the reasons for that?**					
	Your physician has had extensive training and will make the best decisions for you	121/150 (81)	37/42 (88)	58/73 (80)	26/35 (74)
	You have a good relationship with your physician	84/150 (56)	22/42 (52)	44/73 (60)	18/35 (51)
	Your physician can explain information better	67/150 (45)	15/42 (36)	36/73 (49)	16/35 (46)
**If you trust advice from the Internet more, what are the reasons for that?**					
	More comprehensive	1/3 (33)	0/1 (0)	0/1 (0)	1/1 (100)
	More up-to-date	1/3 (33)	0/1 (0)	0/1 (0)	1/1 (100)
**Did your physician’s recommendation conflict with what you found on the Internet?**					
	No	105/144 (73)	30/40 (75)	48/69 (70)	27/35 (77)
	Sometimes yes, sometimes no	35/144 (24)	9/40 (23)	18/69 (26)	8/35 (23)
	Yes	4/144 (3)	1/40 (3)	3/69 (4)	0/35 (0)
**Did you ask your physician for recommendations for cancer-related websites?**					
	No	142/165 (86)	41/47 (87)	66/81 (82)	35/37 (95)
	Yes	23/165 (14)	6/47 (13)	15/81(19)	2/37 (5)

Based on the median scores of trust measured on the six-point Likert scales ranging from 0 (“no trust”) to 5 (“complete trust”) in this anonymous survey, CIUs trusted referring and Phase I physicians more than Web-based cancer-related information (5.0 vs 3.0, *P*<.001 for both; Figure 1). In general, CIUs’ trust in their referring or Phase I physician and CIUs’ trust in Web-based cancer information did not vary by the specific websites that CIUs reported using to learn about cancer or clinical trials (*P*>.05). However, CIUs who reported visiting the NCI website to learn about cancer reported higher levels of trust in Web-based cancer-related information than CIUs who did not use the NCI website (median=4.0 for both, but a significant difference between groups based on the Mann-Whitney U test: Mann-Whitney U=2576; *P*=.02). Also, CIUs’ trust in their referring or phase I physician did not vary by whether the internet had made them aware of information on their cancer, clinical trials, side effects of treatment, management of symptoms, prognosis for their cancer, or new or alternative treatments (*P*>.05).

Nearly 97% (158/163) of CIUs indicated that they trusted their physician’s advice over the information on the internet (Table 3). Almost all CIUs (150/153, 98% of CIUs) reported that internet-derived information did not cause conflict between them and their treating physician. Also, almost all CIUs (157/158, 99%) indicated that information on the internet did not cause them to ignore their doctor’s advice. Approximately 73% (105/144) of CIUs indicated that their physician’s recommendation did not conflict with what they found on the internet, whereas 24% (35/144) reported at least occasional differences between their physician’s advice and Web-based information. Only 14% (23/165) of CIUs had asked their physician for recommendations for cancer-related websites.

### Factors Patients Use to Determine the Trustworthiness of Websites

CIUs indicated that their means for determining the trustworthiness of websites providing medical information related to their disease were the reputation of the website (90/152, 59%), recommendation of physician (77/152, 51%), and credentials of website authors (69/152, 45%). When asked about factors that they valued most on cancer-related websites, Phase I clinic CIUs cited trustworthiness (96/157, 61%), ease of understanding (86/157, 55%), ease of navigation (69/157, 44%), and physician recommendation (59/157, 38%). When asked “Compared to websites with advertisements, how much would you trust websites without advertisements?”, 50% (80/160) of CIUs indicated “about the same”, 39% (66/160) indicated “more”, and 9% (14/160) indicated “less”.

## Discussion

### Principal Findings

To our knowledge, this is the first analysis of the impact of cancer-related internet use on the patient-physician relationship among patients with advanced cancer in an early drug development clinic. A very high percentage of early-phase CIU patients used the internet for information. We found that, despite the prevalence of the internet, social media, and smartphone apps, early drug development clinic patients relied most on physicians as a source of information on cancer and clinical trials. We also found that CIUs reported high trust in their physicians. Approximately half of the CIUs discussed or planned to discuss internet information with their physicians. Few CIUs asked physicians to recommend websites.

Despite the ubiquity of the internet, CIUs in the early drug development clinic still relied on physicians for information about clinical trials and cancer. Early drug development clinic patients also reported a high level of trust in their referring and Phase I clinical trial physicians. This is congruent with findings in cancer survivors [19] the general population of adults [8,20], patients with breast cancer [21], and patients with early-stage papillary thyroid cancer [22], suggesting that patients have a deep level of trust in their physicians and that physicians are the preferred and most trusted source of health information.

The finding that 42% (72/171) of CIUs used the internet, in part, to select the institution in which they would investigate or pursue cancer care options suggests the potential of internet use and cancer center marketing in influencing health care utilization. By contrast, few patients (23/165, 14%) sought physician recommendations for cancer-related websites. We may be missing an opportunity to educate patients by recommending additional informative websites with clinical trial information, especially in an era of ever-shorter clinic visits. This is also reflected in our finding that physician recommendation was a common reason for trusting a website—if physicians did recommend specific websites, the recommendation(s) would likely influence patient behavior.

Although most patients reported that their physician’s recommendation did not conflict with the information they found on the internet, nearly 1 in 4 patients reported at least occasional conflict. This is particularly important given that the quality of internet-based oncology and clinical trial information may vary greatly [23,24]. As some internet sources may be based on hearsay and may not be well-resourced, it might be helpful to encourage physicians or members of the clinical team to discuss what the patient has seen on the internet and help clarify any discrepancies. It may also be helpful for providers to point patients to trustworthy cancer-related websites. Cancer-related organizations, such as the American Society for Clinical Oncology, the American Association for Cancer Research, and governmental institutes such as the NCI, should consider augmenting regularly updated links to recommended websites with trustworthy cancer and clinical trial information and educating physicians so that they can better guide their patients.

### Strengths and Limitations

This study offered several insights into the impact of cancer-related internet use on the doctor-patient relationship among early drug development clinic patients. Its strengths are that the questionnaire incorporated input from both oncologists and patients in the early drug development clinic setting and that it was pilot-tested among early drug development clinic patients. Limitations include that data on the number of patients who were approached and the number of patients who declined to participate were not collected, and therefore, the response rate cannot be calculated. A possible limitation is that this is a single-center study and that our Phase I clinical trials unit is well-recognized for its early-phase trials. Thus, our study population may be quite unique in that patients may come to MD Anderson’s Phase I unit because of the availability of clinical trials. Whether similar results would be observed in other institutions conducting early-phase trials should be investigated in future studies.

### Conclusion

In conclusion, the results suggest that despite the large number of websites related to cancer and cancer clinical trials, patients in early-phase clinical trial settings still trust their physicians for cancer or clinical trial information over information found on the internet. Cancer-related organizations should consider augmenting regularly updated links to trustworthy, verified websites with cancer and clinical trial information and should educate physicians so that they can better direct their patients to appropriate internet content.

## Appendix

Multimedia Appendix 1 Sources of information on cancer and cancer treatment (including clinical trials) for early drug development clinic patients (N=291).

## Abbreviations

CCTT: Clinical Center for Targeted Therapy
CIU: cancer-related internet users
NCI: National Cancer Institute
