# Supplementary material for: Cancer-Related Internet Use and Its Association With Patient Decision Making and Trust in Physicians Among Patients in an Early Drug Development Clinic: A Questionnaire-Based Cross-Sectional Observational Study
Source: J Med Internet Res. 2019 Mar 14;21(3):e10348. doi: 10.2196/10348 (PMC6437608; doi:10.2196/10348)

**Multimedia Appendix 1.** Sources of information on cancer and cancer treatment (including clinical trials) for early drug development clinic patients (N=291).

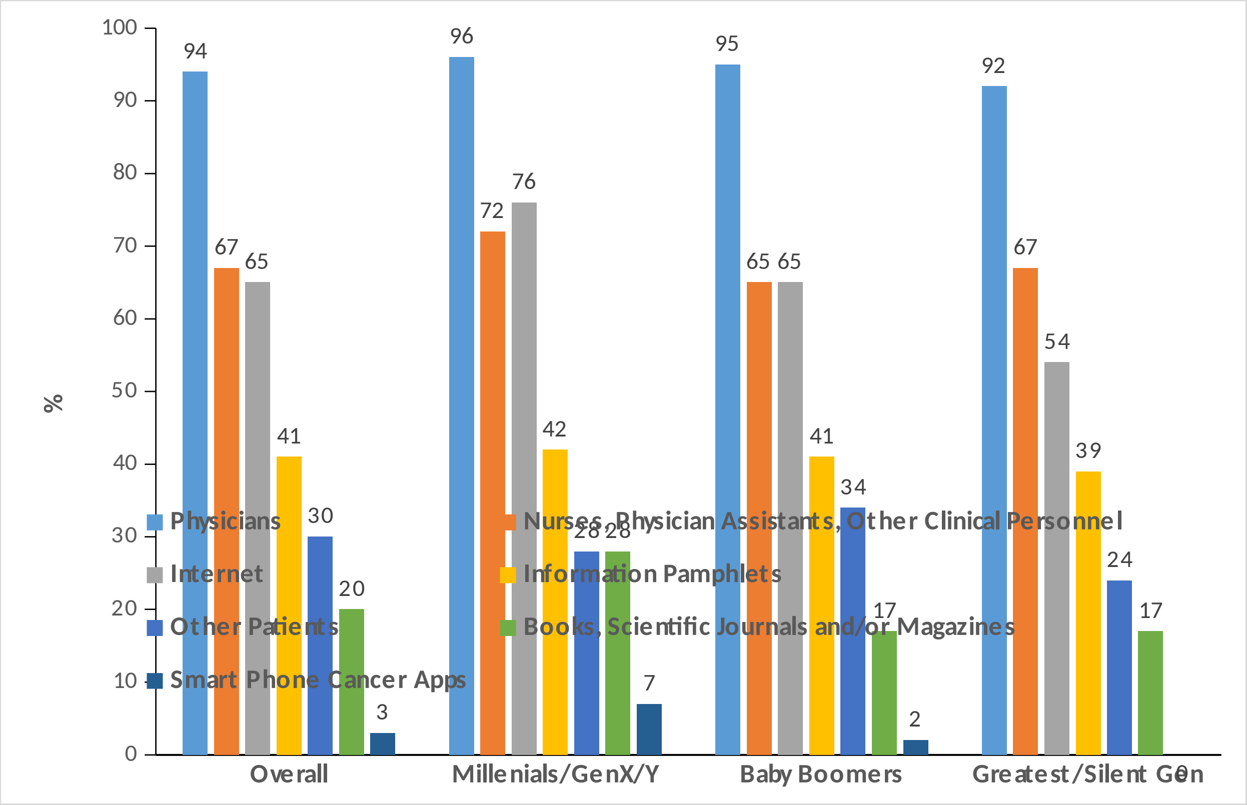

Supplement: Multimedia Appendix 1 [file jmir_v21i3e10348_app1.pdf]
